# Beyond Diagnosis: Exploring Residual Autonomy in Dementia Through a Systematic Review

**DOI:** 10.3390/medicina61050895

**Published:** 2025-05-14

**Authors:** Anna Anselmo, Francesco Corallo, Maria Pagano, Davide Cardile, Angela Marra, Giuseppa Maresca, Rosaria De Luca, Antonella Alagna, Angelo Quartarone, Rocco Salvatore Calabrò, Irene Cappadona

**Affiliations:** IRCCS Centro Neurolesi Bonino-Pulejo, S.S. 113 Via Palermo, C.da Casazza, 98124 Messina, Italy; anna.anselmo@irccsme.it (A.A.); rosaria.deluca@irccsme.it (R.D.L.);

**Keywords:** dementia, activities of daily living (ADL), autonomy, context

## Abstract

*Background and Objectives*: The connection between cognitive decline and autonomy represents a complex and multifactorial area of research. Cognitive decline manifests as a progressive impairment of higher mental functions and is typical of neurodegenerative conditions such as dementia. Autonomy, on the other hand, is defined as an individual’s ability to independently manage activities of daily living and make informed decisions. The objective of this study was to investigate whether certain daily living skills can persist despite the advancement of dementia, and what factors contribute to their preservation in maintaining autonomy. *Materials and Methods*: A literature review was conducted using the databases PubMed, Web of Science, Scopus, Cochrane Library, Embase, and PsycInfo. Out of an initial pool of 12,113 studies, only 19 met the inclusion criteria and were selected for analysis. *Results:* The findings indicate that, in non-institutionalized settings, some daily living abilities may remain preserved despite cognitive deterioration. In contrast, within institutionalized environments, a significant correlation emerged between cognitive decline and the progressive loss of personal autonomy. *Conclusions*: This study highlights the importance of assessing residual abilities in individuals with dementia. Recognizing and supporting these remaining skills can play a crucial role in enhancing quality of life, delaying institutionalization, and promoting autonomy even in the presence of advanced cognitive decline.

## 1. Introduction

### 1.1. Cognition and Autonomy: A Complex Relationship

The relationship between cognitive decline and autonomy is multifaceted and influenced by numerous factors. Cognitive decline typically denotes a reduction in mental functions such as memory, attention, reasoning, and decision making, which may result from aging or specific medical conditions like dementia [1]. In contrast, autonomy encompasses the capacity to manage one’s daily life, make independent decisions, and maintain independence in activities [2]. The effect of cognitive decline on personal autonomy can vary widely among individuals. While some may retain a high degree of autonomy with suitable support, others may require more extensive assistance [3]. Family involvement and early accurate diagnosis of dementia play a critical role, as they significantly affect access to resources, prognosis, and advance planning. The early adaptation of the environment to suit the needs of individuals with dementia can help support their autonomy [4].

### 1.2. ADLs as a Measure of Functional Autonomy

Evaluating the ability to perform activities of daily living (ADLs) is essential in elderly care, particularly for those with cognitive decline such as dementia [5]. As a result, autonomy and its application have become central in elderly care practices. A person-centered approach to care emphasizes collaboration with individuals living with dementia and their family caregivers to personalize care according to clinical needs and improve quality of life (QoL) [6,7]. Within the framework of person-centered care [8], autonomy is linked to QoL and a sense of well-being. The motivation for autonomy often grows stronger when individuals encounter threats to their independence. [9]. The decline in the ability to carry out ADLs is strongly influenced by the severity of dementia, and functional deterioration is a key factor in diminishing QoL for those affected at any stage of the condition [10]. Assessing ADL performance involves understanding not only what individuals are capable of, but also what they actually do in their daily lives. This distinction is important, as individuals may still have the skills but require support or environmental adaptations to utilize them fully. Misjudging this discrepancy can hinder the accurate understanding of remaining capabilities and affect their QoL [11].

### 1.3. ADL Classification and Progression of Decline

ADLs are categorized into basic ADLs (BADLs), more complex instrumental ADLs (IADLs), and advanced or leisure ADLs (AADLs) [10]. Patterns of impairment in ADL performance vary, even in the early stages of dementia. Some individuals may demonstrate relatively strong functional abilities despite significant cognitive decline. ADL assessments are, therefore, valuable tools for evaluating functional levels and monitoring disease progression [12]. Notably, certain ADL skills may remain intact despite disease advancement [13]. Impairments in IADLs typically emerge during mild cognitive decline and the early stages of dementia, whereas BADLs generally deteriorate in the more advanced stages. Nonetheless, individuals with severe cognitive impairments have sometimes retained the ability to carry out specific BADL tasks [14]. These findings underscore the importance of analyzing each domain of BADL independently, as impairment progression varies with cognitive decline severity. Evaluating ADLs in individuals with dementia is further complicated by variations in personal routines and preferences [15]. While some functions may be lost early, others may persist into the middle or late stages of the disease.

### 1.4. Study Aims and Influencing Factors

Understanding which abilities are preserved can clarify the disease’s natural course and aid in optimizing care strategies, thereby enhancing QoL, minimizing hospitalizations, and delaying institutionalization, which is closely tied to increasing dependency [16]. Beyond dementia, depression also contributes to functional impairments in older adults [17]. Symptoms of depression are known to correlate with declines in ADL abilities, both in elderly individuals without dementia and in those with mild dementia [18]. This study aims to explore which daily life skills may remain preserved as dementia progresses and to identify the factors that influence this preservation [19]. Such insights can support the evaluation of individual differences in people with dementia and help identify both risk and protective factors for sustaining ADLs and maintaining autonomy.

## 2. Materials and Methods

A review of the existing literature was carried out to examine the activities of daily living that remain preserved in individuals with dementia, as well as the risk factors that may influence either their preservation or eventual decline. This systematic review was conducted following the guidelines outlined in the PRISMA 2020 framework.

### 2.1. Search Strategy and Inclusion Criteria

The literature search was performed across multiple databases, including PubMed, Web of Science, Cochrane Library, and Scopus, covering articles published up to May 8, 2025, with no additional time restrictions. The following keywords were used: (dementia) OR (Alzheimer) AND (mini-mental state examination) OR (Alzheimer Disease Assessment Scale) OR (Milan Overall Dementia Assessment) OR (Clinical Dementia Rating Scale) AND (activities of daily living) AND (measurement).

To be eligible, studies needed to explore or describe activities of daily living in individuals with dementia and investigate any related risk factors impacting their autonomy. Inclusion criteria were (i) original or protocol studies of any type, and (ii) articles written in English and published in indexed peer-reviewed journals. Only human studies were considered.

Exclusion criteria included studies involving healthy individuals or those with clinical conditions unrelated to diagnosed cognitive decline, as well as articles lacking data on autonomy and daily living activities in dementia. Additionally, systematic, integrative, and narrative reviews, case reports, theses, comments, letters, and editorials were excluded. Reference lists of included studies were also screened, and relevant articles were added if appropriate. No restrictions were imposed on publication year.

### 2.2. Study Selection and Quality Assessment

The selection process involved three main phases. First, non-English articles and duplicates were removed. Then, titles and abstracts were screened for relevance and compliance with the inclusion and exclusion criteria. Finally, full-text versions of the remaining studies were reviewed in detail to confirm eligibility and ensure data completeness.

The quality of the studies included in this review was assessed using the Joanna Briggs Institute’s (JBI) checklists [20]. The systematic review was registered on OSF.io and is accessible at the following link: https://doi.org/10.17605/OSF.IO/FZTGV (accessed on 24 October 2024).

### 2.3. Measurement

The MMSE (Mini-Mental State Examination) scores range from 0 to 30, with a cut-off of 24 indicating possible cognitive impairment. Scores are typically interpreted as follows: 26–30 normal, 21–25 mild impairment, 18–20 moderate impairment, and below 18 severe impairment.

The Katz ADL assesses 6 basic functions, with scores from 0 to 6; a score ≤ 4 indicates significant dependence. The Lawton IADL measures 8 instrumental activities, with scores from 0 to 8; scores < 8 indicate impaired autonomy.

The MODA scale has a total score from 0 to 36, with the following cut-offs: 0–10 minimal impairment, 11–20 mild, 21–30 moderate, 31–36 severe. The cut-off for the diagnosis of dementia is generally set between 20 and 22 points.

The ADAS-Cog has a total score from 0 to 70, with the following cut-offs: 0–10 normal, 11–20 mild impairment, 21–30 moderate, 31–40 severe, and 41–70 severe cognitive impairment. The diagnostic cut-off for Alzheimer’s dementia is generally set between 18 and 20 points.

The Clinical Dementia Rating (CDR) assesses the degree of dementia on a scale from 0 (normal) to 3 (severe), based on six cognitive and functional domains. Scores of 0, 0.5, 1, 2, and 3 indicate no, very mild, mild, moderate, and severe impairment, respectively.

## 3. Results

**Figure 1:**
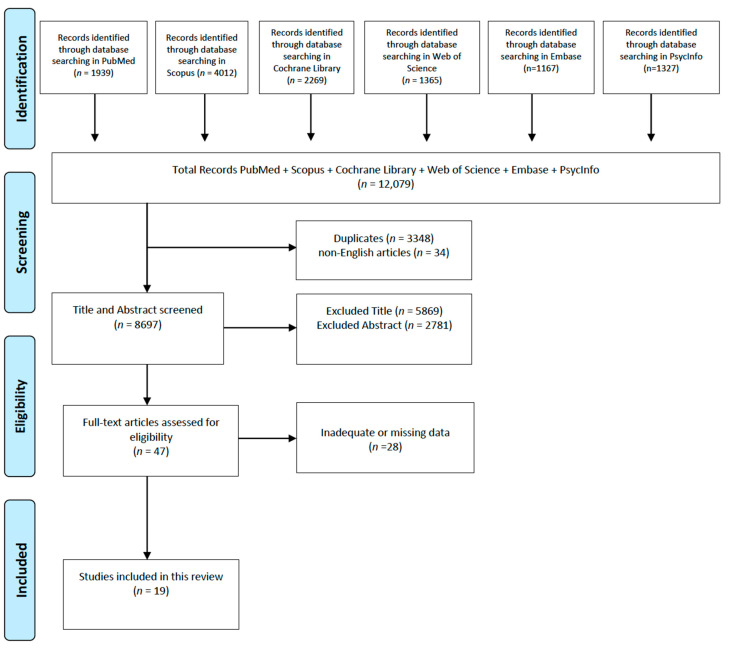
Graph of identification, screening, eligibility, and inclusion of review articles.

The initial electronic search across PubMed, Scopus, Cochrane, and Web of Science yielded a total of 12,113 potentially relevant studies. After removing duplicates, titles, abstracts, and full texts were screened for relevance and eligibility. Following this selection process, 19 articles met the inclusion criteria and were included in the final analysis (Figure 1).

For a detailed description of the studies, see Table 1.

All 14 studies explored the relationship between dementia, the ability to perform activities of daily living (ADLs), and the preservation of autonomy. Among these, nine studies [13,14,16,18,25,26,27,28,29] focused on non-institutionalized settings, while five [19,21,22,23,24] addressed institutionalized environments.

### 3.1. Findings in Non-Institutionalized Settings

The nine studies examining individuals with dementia in non-institutionalized contexts reported varying degrees of preserved daily living abilities despite cognitive decline. Gillioz et al. [16] analyzed individuals with advanced Alzheimer’s disease using the Severe Impairment Battery (SIB) and the Mini-Mental State Examination (MMSE), reporting mean scores of 7.0 ± 2.2 (MMSE) and 69.7 ± 16.9 (SIB). They found that 6.5% were fully independent in basic ADLs (BADLs), with 71% independent in mobility, 53.2% in feeding, 46.8% in bathroom use, and 41.1% in continence. Takechi et al. [18] similarly observed that ambulation and feeding were notably preserved, even as other ADLs declined. Shimokihara et al. [14], using the MMSE and the PADA-D tool, found high levels of preserved autonomy across several areas: feeding (94.8%), bathroom use (87.2%), dressing (73.6%), personal care (65.2%), mobility (81.4%), and bathroom care (72.8%). Andersen et al. [25] examined the impact of dementia severity on independence and quality of life. Among their sample, 57.4% had mild, 30.4% moderate, and 12.3% severe cognitive decline. Despite this, 84% were still independent in performing ADLs. Ikezaki et al. [13] evaluated individuals with mild Alzheimer’s (MMSE ≥ 21) and identified apathy as a key factor in IADL difficulties. Nonetheless, many IADLs remained preserved: among men, 95.8% could use the telephone, 94.4% used transportation, and 94.4% managed finances. Among women, 99.4% retained telephone use, 91.8% household management, 83.6% laundry and transportation, and 90.6% financial tasks. Tabira et al. [26] confirmed that certain instrumental ADLs—such as managing transportation, finances, telephone use, and medication—remained intact despite cognitive impairment.

Potashman et al. [27] found that approximately two-thirds of individuals with mild cognitive impairment (MMSE = 27.7) were able to perform ADLs without supervision, although some of the initial ceiling effects diminished over time.

Liu et al. [28] reported that most individuals with mild dementia (CDR 1.0) retained autonomy in basic ADLs (BADLs) and some domestic instrumental ADLs (IADLs).

Giebel et al. [29] documented that activities such as bathroom use, transfer, and feeding remained relatively preserved across all stages of dementia.

These findings suggest that, in community settings, individuals with dementia often retain the ability to perform a number of essential daily activities.

### 3.2. Findings in Institutionalized Settings

In contrast, studies conducted in institutionalized environments found a more direct association between cognitive decline and the loss of autonomy in ADLs.

Aske et al. [21] reported a significant negative correlation between MMSE scores and ADL performance in a rehabilitation center, highlighting the progressive nature of functional impairment with advancing dementia. Tanaka et al. [22] found that, among hospitalized patients with varying levels of dementia severity, those with more advanced cognitive impairment—measured by the Cognitive Test for Severe Dementia (CTSD)—demonstrated a marked decrease in autonomy over a six-month period. Ashizawa et al. [23], using MMSE and the Barthel Index in elderly care facilities, confirmed that greater dementia severity was associated with poorer ADL performance and diminished quality of life. Riccio et al. [24] also observed that residents with lower MMSE scores were significantly more dependent in their daily activities. The sample comprised individuals diagnosed with Alzheimer’s disease and other types of dementia, categorized into mild, moderate, and severe stages. Finally, Votruba et al. [19] examined how self-reported depressive symptoms, measured via the Geriatric Depression Scale alongside MMSE, influenced IADL performance in people with mild Alzheimer’s. They found that depression further impaired functional autonomy, especially in complex tasks. Collectively, these studies underscore the strong link between cognitive impairment and the loss of autonomy in institutionalized care. The progressive decline in ADL capacity not only limits personal independence, but also has a detrimental impact on overall quality of life (QoL). The results are summarized in the Table 2.

## 4. Discussion

In non-institutionalized settings, individuals with dementia often retain key daily living skills despite cognitive decline, particularly in mobility, feeding, and basic self-care. Several studies reported high levels of independence in both basic and instrumental ADLs, with tasks like telephone use, transportation, and financial management frequently preserved. In contrast, institutionalized individuals showed a stronger correlation between dementia severity and reduced ADL performance, with greater dependency and a diminished quality of life. Depression further exacerbated functional decline, especially in more cognitively demanding activities.

### 4.1. Influence of Living Context on Autonomy and Cognitive Functioning

Our study examined the correlation between living environment, cognitive functioning, and autonomy in activities of daily living (ADLs). The findings show that individuals in institutionalized settings experience a stronger association between cognitive decline and reduced autonomy. In contrast, those in non-institutionalized environments preserve essential skills such as locomotion [18], nutrition [14,16], personal hygiene, transportation use [25], financial management [13], and medication use [26].

Even with dementia, not all abilities decline uniformly. Some individuals can still manage tasks like personal care [30] while struggling with memory or financial responsibilities [31]. Institutionalization is often linked with greater dependency [32], and deprived opportunities to use residual skills may accelerate decline [33]. In contrast, supportive living environments, including innovative housing like dementia villages and group homes, have been shown to maintain autonomy by encouraging daily engagement and social participation [34].

### 4.2. Variability in Functional Decline and Limitations of Standardized Assessment

Functional and cognitive decline in dementia is heterogeneous and nonlinear. Some abilities, such as using the bathroom or feeding, are retained longer, even in severe stages [33]. Evidence shows that social interaction and name recognition can persist even with MMSE scores below 5 [34], and certain cognitive capacities may remain even with an MMSE of 0 [35]. However, standardized tools like the MMSE do not capture this variability well. MMSE scores, although predictive of performance [36], often fail to account for individual strengths. Some studies even show no significant relationship between MMSE scores and functional abilities [37,38]. Consequently, performance-based assessments are recommended to better reflect real-world capabilities [39]. Subjective evaluations are prone to bias, particularly when informants or evaluators underestimate the patient’s actual potential [40]. Functional loss might also be attributed to factors like apathy, depression, or other medical conditions, making diagnosis and assessment more complex [41].

### 4.3. Psychological and Social Determinants of Autonomy

The psychosocial environment significantly affects autonomy in individuals with dementia. Negative attitudes and poor social interactions can damage self-image and cooperation [42,43]. When autonomy is not supported, individuals become less motivated and more apathetic, further diminishing their functionality [30]. These psychosocial losses are strong predictors of reduced quality of life. Apathy and depression, though overlapping, have distinct effects [44]. Apathy is linked to planning and initiating IADLs, while depression affects motivation [45,46]. Both conditions can obscure true functional abilities, leading to underestimation. Effective evaluation should distinguish between these mental states and actual cognitive deficits to avoid misdiagnosis [47,48,49,50].

Social support, particularly from family and caregivers, plays a protective role. Positive relationships can buffer the effects of cognitive decline, enhancing resilience and function [14,16]. Autonomy is sustained when individuals have the freedom to make daily decisions and engage in meaningful activities, emphasizing the importance of relational autonomy in care settings [51,52,53,54].

### 4.4. The Role of Daily Relationships, Home Support, and Innovative Living Solutions

Daily relationships can either facilitate or hinder autonomy [52], and practices aimed at promoting autonomy are increasingly being developed, such as rehabilitation, which focuses on mitigating the impact of dementia on functioning and cognition. These practices are often shaped by a relationship of trust and continuity between staff and residents [54]. The institutionalization of older adults with dementia represents an additional risk factor for total dependency. In many cases, residential facilities do not adequately assess or preserve the abilities and potential of patients, leading to a decline in their quality of life.

The standardized and routine-based approach of institutions tends to overlook individual abilities, fostering dependency rather than supporting autonomy.

Even within the home environment, it is essential to sustain the residual autonomy of people with dementia. In this regard, psychoeducational sessions for caregivers serve as a valuable tool to provide both practical and emotional support in the daily management of the patient. These programs not only promote strategies that enhance the individual’s remaining abilities, but also help to ease the emotional burden experienced by caregivers, ultimately improving the quality of both care and relationship.

Given the current lack of in-depth knowledge about the features of innovative housing solutions—such as shared housing, eco-farms, dementia villages, and group homes—described as alternatives to traditional nursing homes, future research should explore this area. A better understanding of the potential key elements of these innovative living arrangements is needed to support the autonomy of people with dementia.

### 4.5. Clinical Implications, Limitations, and Future Directions

Our review is the first, to our knowledge, to support the idea that autonomy in daily living can be maintained even with a dementia diagnosis. The key finding is that people living at home tend to preserve more autonomy compared to those in institutional settings due to more individualized support and environmental stimulation.

However, several limitations exist. The study sample was heterogeneous, and results across studies were inconsistent. Many assessments risk underestimating actual abilities due to variability in tools and populations. Further research—particularly systematic reviews and meta-analyses—is needed to clarify the relationship between cognitive decline and preserved abilities. There is a pressing need for a more comprehensive assessment tool combining self-reports, informant input, and real-life performance tasks. In addition, improved assessment protocols in residential care settings are crucial for preventing the development of avoidable dependency. Facilities that fail to consider individual needs contribute to accelerated decline [54,55,56], while others with tailored programs show more favorable outcomes [57,58,59]. The implementation of self-monitoring and quality improvement strategies could promote best practices across all institutions [60,61]. Ultimately, housing models such as shared homes, eco-farms, and dementia villages should be further studied for their potential in supporting autonomy. Expanding innovative person-centered care approaches could greatly enhance the quality of life for individuals living with dementia.

## 5. Conclusions

In conclusion, our study highlights the critical importance of accurately assessing the residual abilities of people with dementia. Recognizing and supporting these abilities can play a critical role in improving quality of life, delaying or avoiding institutionalization, and fostering autonomy despite cognitive decline. There is a clear need to develop more consistent and reliable assessment tools that take into account the full range of risk factors that may obscure or underestimate a person’s true functional potential. For example, future research should focus on refining the assessment tools currently in use through the introduction of more specific and sensitive scales that take into account real residual abilities in activities of daily living, especially at different stages of dementia. In particular, it would be desirable to develop tools that integrate not only self-assessment and caregiver narrative, but also direct observation in ecologically valid environments, such as the home setting or day care centers.

This innovative approach should be central when exploring alternatives to traditional institutional care. By promoting and using residual skills, we can better support the independence and dignity of people with dementia.

## Figures and Tables

**Table 1:** Characteristics of included studies.

References	Objective	Sample	Tools	Context	ADL Results	Dementia Severity	Preserved Autonomies
Aske, 1990 [21]	To compare scores obtained through the Mini-Mental and ADL autonomy scales	37 subjects	MMSE ADL	Rehabilitation center for dementia patients	There is a negative correlation between Mini-Mental and ADL scores. That is, the higher the Mini-Mental score, the lower the ADL score.	Missing	NO autonomies
Votruba et al., 2015 [19]	To investigate the relationship between self-reported depressive symptoms and caregiver-perceived depressive symptoms with ADLs	71 subjects	MMSE WMS-III GDS-15 ADL IADL	Institutionalized	Depressive symptoms were associated with worse performance on IADL measurements.	Mild dementia (mean MMSE = 19.86)	NO autonomies
Tanaka et al., 2020 [22]	To identify clinical factors influencing ADLs at baseline and after 6 months	131 subjects	PSMS MMSE CTSD	Hospital setting (institutionalized)	Only cognitive function assessed by CTSD at baseline was associated with ADLs.	Mild to moderate dementia: 38 patients; severe dementia: 93 patients	NO autonomies
Ashizawa et al., 2021 [23]	To evaluate the impact of Alzheimer’s disease severity on ADLs, QoL, and care costs in Japanese elderly care facilities	287 subjects	EQ-5D-5L BI MMSE	Institutionalized	With worsening AD severity, BI scores significantly decreased.	Mild: 53 patients; moderate: 118 patients; severe: 116 patients	NO autonomies
Riccio et al., 2007 [24]	To assess whether decline in cognitive functions is associated with functional deterioration	47 subjects	ADL IADL MMSE CGA	Institutionalized	MMSE was significantly correlated with dependence in ADLs.	Severe dementia (MMSE 0–9): 5 patients; moderate dementia (MMSE 10–29): 23 patients; mild dementia (MMSE 20–30): 19 patients	NO autonomies
Ikezaki et al., 2020 [13]	To examine the relationship between global cognitive functions, neuropsychiatric symptoms, and IADLs in patients with mild AD	230 subjects	ADAS-Jcog FAB GDS-15 ADL MMSE NPI CDR	Hospital setting (non-institutionalized)	Apathy on NPI was associated with numerous IADL elements. Preserved autonomies in men were phone use (95.8%), transportation (94.4%), financial skills (94.4%). In women, phone use (99.4%), housekeeping (91.8%), laundry (92.5%), transportation (83.6%), financial skills (90.6%).	All patients scored ≥ 21 on MMSE (mild Alzheimer’s)	YES autonomies
Takechi et al., 2012 [18]	To analyze the decline of different types of ADLs (BADL, IADL, and AADL)	39 subjects	MMSE ADL IADL AADL BADL	Hospital setting (non-institutionalized)	Physical ambulation and feeding remained preserved. Mean MMSE score: 22.3 ± 3.4.	Missing	YES autonomies
Andersen et al., 2004 [25]	To identify key factors influencing QoL related to health in dementia patients	244 subjects	CDR MMSE ADL EQ-5D	Non-institutionalized	Overall, 16% classified as dependent, 84% classified as independent in ADL performance.	Mild: 140 (57.4%); moderate: 74 (30.4%); severe: 30 (12.3%)	YES autonomies
Gillioz et al., 2009 [16]	To evaluate characteristics of patients in severe stage of Alzheimer’s disease	126 subjects	MMSE SIB ADL IADL MNA	Non-institutionalized (outpatient)	Overall, 6.5% were totally independent in BADLs, 71% were completely independent in mobility, 53.2% in feeding, 46.8% in toileting, and 41.1% in continence. Mean MMSE score: 7.0 ± 2.2. Mean SIB score: 69.7 ± 16.9.	Missing	YES autonomies
Tabira et al., 2024 [26]	To assess compromised and intact IADLs with severity of cognitive decline in elderly with Alzheimer’s disease	115 subjects	MMSE IADL	Non-institutionalized	Use of transportation means, financial management, telephone use, and medication management were preserved and independent of cognitive decline.	Missing	YES autonomies
Shimokihara et al., 2022 [14]	To clarify characteristics of processes for BADLs with severity of cognitive decline in elderly with dementia	143 subjects	MMSE PADA-D	Non-institutionalized	Some ADLs remained preserved. Feeding: 94.8%. Toileting: 87.2%. Dressing: 73.6%. Personal care: 65.2%. Mobility: 81.4%. Bathing: 72.8%.	Mild: 53 patients; moderate: 73 patients; severe: 17 patients	YES autonomies
Potashman et al., 2023 [27]	To evaluate the measurement properties of ADCS-ADL-MCI in subjects with cognitive decline	769 subjects	ADCS ADL MMSE	Non-institutionalized	At baseline, about two-thirds of the individual items showed ceiling effects, with over 80% of patients performing daily activities “without supervision”. By month 36, most of these ceiling effects persisted, though to a lesser extent, which were no longer evident for items 1, 10, and 11.	Mild cognitive impairment: 58% (MMSE = 27,7)	YES autonomies
Liu et al., 2007 [28]	To explore the activities of daily living ADL performance profile of community-living people with dementia and to investigate its relationship with dementia severity	86 subjects	CDR IADL	Non-institutionalized	Subjects were generally able to perform most basic BADLs, including personal care, feeding, dressing, and using the bathroom. For basic IADLs, most subjects were able to perform basic instrumental daily activities ADLs in the household, such as home maintenance and laundry.	Both the median and mode score of the CDR was 1.0 (range 0.5–3.0), indicating that the majority of the subjects had mild dementia	YES autonomies
Giebel et al., 2014 [29]	Analyze the impact of deterioration in basic daily activities of living at different stages of dementia	1026 subjects	MMSE ADL	Non-institutionalized	Bathroom use, transfer, and feeding remained relatively preserved during all stages of dementia.	Mild dementia (*n* = 263); moderate dementia (*n* = 521); severe dementia (*n* = 242)	YES autonomies

Legend: MMSE: Mini-Mental State Examination; SIB: Severe Impairment Battery; ADL: activities of daily living; IADL: instrumental activities of daily living; NPI: Neuropsychiatric Inventory; MNA: mini-nutritional assessment; PSMS: Physical Self-Maintenance Scale; CTSD: Cognitive Test in Severe Dementia; ADAS-JCOG: Japanese version of the Alzheimer Disease assessment scale cognitive part; FAB: frontal assessment battery; GDS-15: 15-item Geriatric Depression Scale; QoL: quality of life; CDR: Clinical Dementia Rating; AADL: advanced activity of daily living; BADL: basic activities of daily living; EQ-5D: EuroQol-Five Dimension Scale; BI: Barthel Index; WMS-III: Wechsler Memory Scale III; CGA: comprehensive geriatric assessment; PADA-D: Process Analysis of Daily Activity for Dementia (PADA-D); ADCS: Alzheimer’s Disease Cooperative Study.

**Table 2:** Summary of results.

Reference (Author, Year)	Severity of Dementia	Context	Preserved Autonomy
Aske, 1990 [21]	Missing	Institutionalized	No
Andersen et al., 2004 [25]	Mild: 140; moderate: 74; severe: 30	Non-institutionalized	Yes
Riccio et al., 2007 [24]	Severe: 5; moderate: 23; mild: 19	Institutionalized	No
Gillioz et al., 2009 [16]	Severe (mean MMSE = 7.0)	Non-institutionalized	Yes
Takechi et al., 2012 [18]	Missing	Non-institutionalized	Yes
Votruba et al., 2015 [19]	Mild (mean MMSE = 19.86)	Institutionalized	No
Ikezaki et al., 2020 [13]	Mild (MMSE ≥ 21)	Non-institutionalized	Yes
Tanaka et al., 2020 [22]	Mild–moderate: 38; severe: 93	Institutionalized	No
Ashizawa et al., 2021 [23]	Mild: 53; moderate: 118; severe: 116	Institutionalized	No
Shimokihara et al., 2022 [14]	Mild: 53; moderate: 73; severe: 17	Non-institutionalized	Yes
Potashman et al., 2023 [27]	Mild cognitive impairment (58%)	Non-institutionalized	Yes
Tabira et al., 2024 [26]	Missing	Non-institutionalized	Yes
Liu et al., 2007 [28]	Mild dementia	Non institutionalized	Yes
Giebel et al., 2014 [29]	Mild dementia (*n* = 263); moderate dementia (*n* = 521); severe dementia (*n* = 242)	Non institutionalized	Yes

## Data Availability

The data that support the findings of this study are available from the corresponding author upon reasonable request.
